# Brazilian Artisanal Cheeses: Diversity, Microbiological Safety, and Challenges for the Sector

**DOI:** 10.3389/fmicb.2021.666922

**Published:** 2021-04-20

**Authors:** Ana Paulina Arellano Pineda, Gabriela Zampieri Campos, Natan Jesus Pimentel-Filho, Bernadette Dora Gombossy de Melo Franco, Uelinton Manoel Pinto

**Affiliations:** ^1^Department of Food and Experimental Nutrition, Food Research Center, Faculty of Pharmaceutical Sciences, University of São Paulo, São Paulo, Brazil; ^2^Center for Natural Sciences, Federal University of São Carlos, Campus Lagoa do Sino, São Paulo, Brazil

**Keywords:** food safety, ripening, foodborne pathogens, fermentation, good manufacturing practice

## Abstract

Artisanal cheeses made with raw milk are highly appreciated products in Brazil. Most of these cheeses are produced in small facilities across different production regions in the country, some of which have been granted a protected designation of origin and are award winners. The most prominent state that manufactures these products is Minas Gerais (MG), but production is also gaining strength in other Brazilian states. The major challenge faced by artisanal cheese production is related to microbial risks associated with foodborne pathogens when the quality of the raw milk is unsatisfactory. Regulations created for the dairy industry are constantly been revised and adapted, considering the small-scale production of Brazilian artisanal cheeses, in order to guarantee safety at all steps of cheese production and commercialization. This text presents a summary of the huge diversity of artisanal cheeses produced in the country, grouped by geographical regions, and reviews the current challenges faced by producers and government considering the safety of these cheeses.

## Introduction

The history of artisanal cheese production in Brazil dates to the second half of the 18th century. After the arrival of Europeans in Brazil in 1500, Portuguese settlers brought cattle from Serra da Estrela, Portugal, to the region which now corresponds to the state of Bahia, in the Northeastern part of the country. Herds of domesticated cattle expanded southward along the São Francisco River, reaching the region of Serro, in Minas Gerais (MG) state, where gold explorers started the production of artisanal cheeses, using raw bovine milk and rennet from parts of the stomach of calves (Sertão Brás, 2017a). When gold mining and sugar cane exploration lost strength, the manufacture of artisanal cheeses gained economic importance, leveraged by the construction of a highway in 1929–1930 connecting the region of Serro with other municipalities in MG state, and Belo Horizonte, the state’s capital city. A great expansion of the market occurred in the following years, and artisanal cheese production became an autonomous element of the economy, not only for the region, but also for the entire state of Minas Gerais (Pires, 2013a,b). Although having started only recently in several parts of the country, artisanal cheese production is gaining increasing importance as economic income for thousands of families in rural areas, contributing to the local economies. This is especially due to the new demands from consumers who are increasingly seeking differentiated products, less processed, culturally rich and with a unique identity.

**Figure 1:**
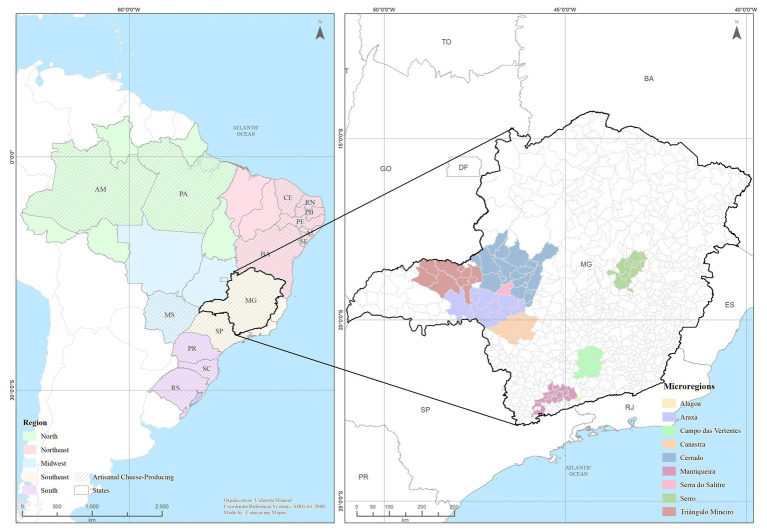
States within each Brazilian region where the main Brazilian artisanal cheeses are produced. **North**: AM, Amazonas; PA, Pará; **Northeast**: AL, Alagoas; BA, Bahia; CE, Ceará; PB, Paraíba; PE, Pernambuco; RN, Rio Grande do Norte; SE, Sergipe; **Southeast**: MG, Minas Gerais; SP, São Paulo; **Central-West**: MS, Mato Grosso do Sul; **South**: PR, Paraná, RS, Rio Grande do Sul; SC, Santa Catarina. The inset highlights the nine artisanal cheese producing microregions in the state of Minas Gerais (MG).

According to the Brazilian Institute of Geography and Statistics (Instituto Brasileiro de Geografia e Estatística - IBGE), Brazil encompasses 26 states and the Federal District, grouped into five geographical regions: Southeast, Northeast, South, North and Central-West (IBGE, 2020). In addition to Minas Gerais in the Southeast region, which is by far the largest cheese producer state, artisanal cheese production is growing rapidly in other states (Figure 1), with cheeses for all tastes and purchasing power. Two recent reviews show complementary aspects of artisanal cheese production in Brazil: Kamimura et al. (2019) characterized the technological, physical-chemical, and microbiological features of the main types of artisanal cheeses in the country, and Camargo et al. (2021) discussed the quality and safety of these products and presented insights into the regulatory aspects of the production chain.

With many research advances, several recent regulatory developments throughout the country and an increased appreciation for artisanal cheese consumption, an updated view of the Brazilian production chain is still required. The current review highlights the diversity of artisanal cheese production in Brazil, discussing aspects that have not been evaluated previously, presenting an updated outlook of artisanal cheese production in the country. We underscore the areas in which more research needs to be conducted and indicate how Brazilian scientists have contributed to advancements in the field. We finally present a perspective for how research and extension collaborative efforts could drive quality and safety improvements in artisanal cheese production.

## Diversity of Artisanal Cheeses Produced in Brazil

Artisanal cheese production is performed by small rural facilities across the nation, with historical, cultural, and technological aspects that are specific to the various producing regions. The best-known types of Brazilian artisanal cheeses, according to the producing region and state, are listed in Table 1. Figure 1 shows an updated view of the geographical location of these producing regions.

**Table 1:** Types of Brazilian artisanal cheeses.

Cheese type	Production region	Production state	Type of milk	Starter culture type	Classification	Ripening time (minimum period, in days)
*Araxá*	Southeast	MG	Cow	Endogenous^*^		14
*Campo das Vertentes*			Cow		Medium moisture	22
*Canastra*			Cow			
*Canastra Real or Canastrão*			Cow		Medium moisture	60
*Cerrado*			Cow			
*Serra do Salitre*			Cow			
*Triângulo Mineiro*			Cow			
*Serro*			Cow			17
*Cabacinha*			Cow	*Endogenous*	Medium to high moisture	15
*Alagoa*			Cow	*Endogenous*	Low moisture	14
*Mantiqueira de Minas*			Cow	*Endogenous*	Low moisture	14
Different Types of Cheese		SP	Goat/Cow/Buffalo/Goat/Sheep	Varies according to the type of cheese		
*Porungo*			Cow	*Endogenous*	High moisture	Not ripened
*Coalho*	Northeast	AL, BA, CE, MA, PB, PE, RN, SE	Cow/Goat/ Buffalo/Sheep	Commercial	Medium to high moisture	Fresh or ripened up to 10 days.
*Manteiga* *(Sertão Cheese)*		CE, PE, RN BA, PB,	Cow/Goat	Endogenous	Medium to high moisture	Not ripened
*Flor de Mandacaru*		PE	Cow	No information available		60
*Cariri*		PB	Goat	Commercial		7
*Dom Ariano*		PB	Goat	No information available		180
*Dom Manelito*			Cow	No information available		120
*Colonial*	South	PR, RS, SC	Cow	Commercial	Medium moisture	10
*Serrano*		SC, RS	Cow	Commercial	Medium moisture	60
*Kochkäse*		SC	Cow	Not used	High moisture	Not ripened
*Marajó*	North	PA	Buffalo/Cow	*Endogenous*	High moisture	Not ripened
*Caipira*	Central West	MS	Cow	*Endogenous*	Medium to low moisture	Up to 60

* Endogenous culture is the fermented whey collected in the manufacture of the cheese from the previous day.

AL, Alagoas; BA, Bahia; CE, Ceará; MG, Minas Gerais; MA, Maranhão; MS, Mato Grosso do Sul; PA, Pará; PB, Paraíba; PE, Pernambuco; PR, Paraná; RN, Rio Grande do Norte; RS, Rio Grande do Sul; SP, São Paulo; SC, Santa Catarina; SE, Sergipe.

### Artisanal Cheeses From the Southeast

#### Cheeses From the State of Minas Gerais

The state of Minas Gerais in Brazil is historically recognized for its secular tradition in cheese making. This state is the largest cheese producing in Brazil and stands out because of the production of a large variety of artisanal cheeses, collectively named as *Minas artisanal cheese*.

The manufacture of *Minas artisanal cheese* started with the Portuguese colonizers in the 18^th^ century, becoming the most popular and consumed artisanal cheese in the country. These products have a great socio-economic importance in the state, as thousands of rural families depend on them for their survival (Meneses, 2006; Bemfeito et al., 2016). These cheeses are conventionally produced in seven spatially limited regions in the state harboring peculiar geomorphological and cultural characteristics: Araxá, Campo das Vertentes, Canastra, Cerrado, Serra do Salitre, Serro, and Triângulo Mineiro (IMA, 2016; Figure 1). The manufacturing procedure in these regions is similar and follows the Portuguese tradition, differing mainly in the curd pressing stage: in Serro, the curd is pressed with bare hands while in Canastra, Serra do Salitre and Cerrado cheese cloth is used. Thus, depending on the pressing method, more whey can be retained in the curd and, consequently, the product will present significant differences in flavor and texture. It is worth mentioning that there are other regions recognized as cheese producers in Minas Gerais, but the production process is carried out differently from the *Minas artisanal cheese* process, as it will be described below (EMATER, 2004; Minas Gerais, 2020a,b).

**Figure 2:**
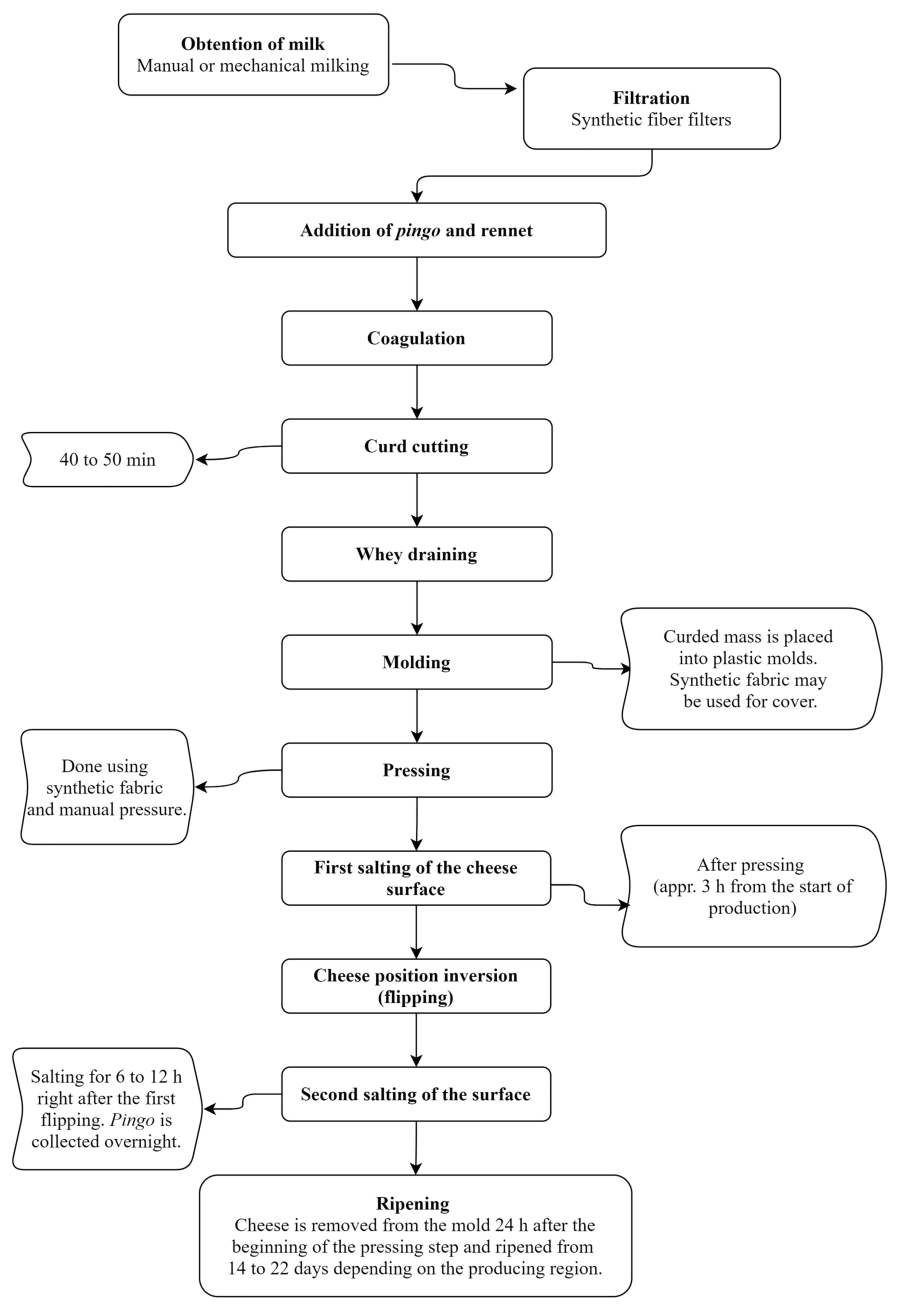
Flowchart of *Minas artisanal cheese* production. *Pingo* is a type of endogenous starter culture composed of fermentative microorganisms. It is collected from freshly manufactured cheeses during the molding stage, and it is used to make the next day’s batch (backslopping method).

Figure 2 shows a flowchart of the production of *Minas artisanal cheese*, the most studied Brazilian artisanal cheese. This product is manufactured with unpasteurized cow milk, added liquid or powdered rennet, salt and a type of endogenous starter culture usually referred to as “*pingo*,” known as the back-slopping method. *Pingo* is composed of fermentative microorganisms, obtained from the whey that drains from freshly manufactured cheeses during the molding stage, and it is used to make the next day’s batch (Perin et al., 2017). The microbial diversity of *pingo* is characteristic of each production region, explaining the unique sensorial characteristics (taste, texture, color, and aroma) that develop during ripening of cheeses produced in these regions (Meneses, 2006).

The Minas Gerais State Law 23157 (Minas Gerais, 2018a) defines *Minas artisanal cheeses* as those manufactured with fresh and raw whole cow milk, harboring specific identity and quality characteristics. Due to the traditional, cultural and economic importance, *Minas artisanal cheeses* were granted with the Cultural Property of Immaterial Nature status, conferred by the National Historic and Artistic Heritage Institute (*Instituto do Patrimônio Histórico e Artístico Nacional* - IPHAN) in 2008 (IPHAN, 2008).

The most relevant types of *Minas artisanal cheeses* are described below:

##### Canastra Cheese

The *Canastra cheese* is a typical product from the Serra da Canastra region, covering Delfinópolis, São Roque de Minas, Vargem Bonita, Tapiraí, Bambuí, Medeiros, Piumhi, São João Batista do Glória and Córrego D’Anta municipalities (Minas Gerais, 2018b). In this region, there are 4,813 dairy farms and approximately 264,000 animals, resulting in an average of 55 animals per farm (EMATER, 2004). Approximately 800 of these farms are dedicated to production of *Canastra cheese*. Average milk production of matrices is about 1,400 liters/lactation, and the fat content is close to 3%, i.e., excellent for cheese production (EMATER, 2004; Meneses, 2006). In 2002, around 60 producers joined efforts and created the Canastra Cheese Producers Association (*Associação dos Produtores de Queijo da Canastra* - APROCAN), aiming at increasing their reach and protecting their products. The total turnover of this group of producers is around R$ 60 million (11 million USD) per year, with an average of 25 cheeses produced every day in each farm (Folhapress, 2019). After the official recognition of APROCAN in 2005, several new producers have joined the association and many others are in the process of joining (Folhapress, 2019).

The *Canastra cheese* has the following characteristics: cylindrical shape, flat or slightly curved at the sides and a slightly acidic and non-spicy flavor. It has a yellowish-white color and a thin yellowish crust that may darken with ripening. The required ripening time is a minimum of 22 days, resulting in a semi-hard or slightly soft, buttery and compact cheese (APROCAN, 2011). There are some variants of *Canastra cheese*:

- *Traditional*: cheeses produced in bottomless cylindrical containers, presenting 6–9 cm height, 17 cm diameter, 900–1,300 g weight, and 22 days of ripening (APROCAN, 2011; Borges et al., 2019).
- *Merendeiro*: smaller cheeses, presenting 10 cm diameter, 6 cm height, 300–400 g weight, and 22 days of ripening (APROCAN, 2011).
- *Real* (also called *Canastrão*): larger in size (28–35 cm diameter and 10–18 cm height), these cheeses have 5,000–7,000 g weight and are ripened for at least 60 days (APROCAN, 2011, 2014). Their flavor is sweet and light, with a bitter taste at the end, reminiscent of Dutch cheeses. According to the local memory, this type of *Canastra cheese* was formerly produced for special occasions, such as visits of authorities from the church, the government or the military. The main characteristic of *Canastra Real* is the presence of propionic bacteria, responsible for the curing process, and production of gas, which contributes to the puffiness and formation of round holes in the cheese, similar to Emmental and Gruyère cheeses.

In May 2008, the *Canastra cheese* was recognized as a Brazilian Intangible Cultural Heritage (IPHAN, 2008). In 2012, the National Institute of Industrial Property (*Instituto Nacional de Propriedade Industrial* - INPI) granted these cheeses with the “Geographical Indication” seal (SEBRAE, 2018; INPI, 2019) and the Serra da Canastra region was recognized as a reference in production of cheeses (EMBRAPA, 2019a). *Canastra cheeses* are gaining international recognition, winning many international awards, such as the 24 super gold, gold, silver and bronze medals in the “*Mondial du Fromage et des Produits Laitiers*” competition, in France, in June 2019 (EM, 2015; G1, 2019).

The unique features of *Canastra cheese* can be attributed to the milk that comes from mixed-breed of *Bos taurus*, *Bos indicus*, and other variants and to the altitude and characteristic climate, in addition to natural pastures for cattle feeding, comprised by *Capim meloso* (*Melinis minutiflora*) and native grasses, which are being gradually replaced by more productive ones, such as *Brachiaria* spp. and *Panicum* (IMA, 2013; Meneses, 2006).

##### Serro Artisanal Cheese

This type of cheese corresponds to a group of cheeses produced by approximately 760 cheese producers, located in the municipalities of Alvoradas de Minas, Conceição do Mato Dentro, Dom Joaquim, Materlândia, Paulistas, Rio Vermelho, Sabinópolis, Santo Antônio do Itambé, Serra Azul de Minas, Coluna and Serro. The average daily production in Serro and neighboring municipalities is around 10,000 cheeses, with a volume of 110 L of milk per property (Sertão Brás, 2017b).

According to Minas Gerais State Ordinance 1305/2013 (Minas Gerais, 2013), *Serro cheese* must be ripened for 17 days, resulting in products with firm consistency and mild and slightly acid flavor. They present a thin crust and a yellowish white color and a natural sheen. The shape is cylindrical, with 13–15 cm diameter and 4–6 cm height and weight varies from 700 to 1,000 g (EMATER, 2003). *Serro cheeses* have also been recognized as a Brazilian Intangible Cultural Heritage in 2008 (Minas Gerais, 2018a). In 2011, INPI granted the “Geographical Indication” seal in the item of “Origin Indication” (SEBRAE, 2018).

The peculiar sensorial properties of *Serro cheeses* are devoted to milk coming from European cow breeds such as Dutch, Jersey and Swiss Pardo. The Serro region has about 124,000 animals, distributed in 2,581 properties, usually small cattle rancher families, with an average of 50 animals per property (EMATER, 2002). Average production is 110 L of milk per day, per property. It is estimated that *Serro cheese* is produced in 6,000 properties, but only 756 are registered in this activity, where 10,000 pieces are produced per day. The cattle in this region is fed with *Brachiaria* spp., *Panicuns*, Jaraguá grass (*Hyparrhenia rufa*), Meloso grass (*Melinis multiflora*), and leguminous plants such as Carrapicho (*Cenchrus echinatus* L.), Beiço de boi (*Desmodium* sp.), Calopogônio (*Calopogonium mucunoides*), among others (APROCAN, 2014).

##### Araxá Artisanal Cheese

The *Araxá cheese* is a typical product from the micro-region comprising the municipalities of Araxá, Tapira, Pratinha, Conquista, Ibiá, Campos Altos, Perdizes, Pedrinópolis and Sacramento. These cheeses present semi-hard consistency, with a tendency to soft, and a butter-like nature. They have a thin crust and are yellowish without cracks and generally present cylindrical size of 13–15 cm diameter and weigh between 1.0 and 1.2 kg. The smell and taste are acid, pleasant and not spicy (EMATER, 2003).

The local herd for milk production is composed mostly by mixed-breed animals, mainly Dutch, with around 476,000 animals, divided into roughly 7,000 farms, with an average of less than 60 heads per farm. The average production is 2,400 L of milk per lactation, with fat content above 3.0%. Around 88% of the area is pasture lands, and the remaining has unmodified vegetation cover (EMATER, 2003). Physical and environmental conditions such as altitude, soil and microclimate, provide special pastures, mainly *Capim gordura* (also known as *Capim meloso*), *Capim Jaraguá*, and *Macega* (EMATER, 2003). There are approximately 1,336 cheese producers in this micro-region.

##### Other Types of Artisanal Cheese Produced in Minas Gerais State

This group encompasses cheeses named *Parmesão da Mantiqueira* (also known as *Queijo Artesanal Mantiqueira de Minas*), *Parmesão de Alagoa* (also known as *Queijo Artesanal de Alagoa*), *Cabacinha* and *Requeijão Moreno*. The region of Mantiqueira and the municipality of Alagoa were recently recognized as artisanal cheese producer regions in the state of Minas Gerais (Minas Gerais, 2020a,b; Figure 1).

Despite being produced with raw milk, *Parmesão da Mantiqueira* and *Parmesão de Alagoa* are different from the traditional *Minas artisanal cheeses* because they are made with commercial starter culture and are submitted to a thermal process during production (Minas Gerais, 2019; SEAPA, 2020).

The *Cabacinha cheese*, a local version of the Italian Caciocavallo cheese, is produced in the Vale do Jequitinhonha, in the North of the state. The curd is cooked in boiling water, shaped into natural gourd or teardrop shape, tied in pairs by strings and hung to dry. Due to its peculiar shape that reminds the Brazilian porongo (or porungo) fruit (*Lagenaria siceraria*), the *Cabacinha cheese* is also known as *Porongo cheese* (or *Porungo cheese*). The cheese can be smoked or filled with butter (Filho et al., 2017; Vasek and Filho, 2019).

The *Requeijão Moreno cheese* is mechanically pressed, with high salt content (EM, 2018; Sobre Queijos, 2020). This cheese is produced in Jequitinhonha and Mucuri Valleys, located in the North of Minas Gerais.

##### Moldy Artisanal Cheeses

Production of moldy cheeses, similar to the French Brie cheese, is gaining importance in the state of Minas Gerais, particularly in Serra da Canastra and Serro. These cheeses present a white mold rind, are firm to the touch but creamy in the mouth, with striking aroma and taste that can be delicate or intense (EM, 2018). Fungi that proliferate in the surface of the cheese introducing their digestive enzymes into the curd, breaking down fat and proteins, turning it softer (Hui et al., 2004). Even though Brazilian artisanal moldy cheeses do not have a specific legislation, they have gained a precious status in specialized stores and fine restaurants in several Brazilian big cities. Little information is available on the production techniques of artisanal moldy cheeses in Brazil (Tristão, 2015), but it is known that the fungi come from the ripening rooms, as producers do not add a specific mold to their product. The production conditions are not controlled and the contamination, in a way, occurs at random.

#### Cheeses From the State of São Paulo

Artisanal cheeses produced in the state of São Paulo are distinct from those of the Minas Gerais state. As producers in the state of São Paulo cannot rely on centuries of tradition, they invest in innovation, using European fine cheeses as models. However, little is known about the production processes, annual turnover, market, and production practices. Even the “artisanal” concept in the state of São Paulo differs from that used in Minas Gerais state for raw milk cheeses. In São Paulo, this nomenclature refers to cheeses produced in small producing properties, using a large array of technological processes and ingredients, some of them quite sophisticated. These cheeses may be produced with raw or pasteurized milk from different types of animals (cow, goat, buffalo, and sheep), resulting in unique cheeses not found in other parts of the country. There is no current legislation that specifically deals with artisanal cheese production in the state of Sao Paulo and research is needed to characterize the products and production processes.

Aiming at strengthening the artisanal cheese sector in the state of Sao Paulo, and removing these cheeses from clandestinity, a group of local producers created, in 2017, the São Paulo Association of Artisanal Cheese (*Associação Paulista do Queijo Artesanal* - APQA). Currently, the APQA affiliates around 80 cheese producers from across the state (Pereira, 2018). APQA includes not only producers with more than 20 years of history in cheese production, but also new cheese makers starting ventures in the sector.

For the purpose of this review, artisanal cheeses produced in the state of São Paulo were divided into two groups:

##### Cheeses From the Paulista Artisanal Cheese Path (Caminho do Queijo Artesanal Paulista)

Currently, the *Paulista Artisanal Cheese Path* (Caminho do Queijo Artesanal Paulista) comprises 10 cheese producers from the municipalities of Joanópolis, Amparo, Porangaba, Itapetininga, São João da Boa Vista, São José do Rio Pardo, Pardinho, Cabreúva, Bofete and Porto Feliz, in the state of São Paulo. These producers use raw or pasteurized milk from cow, buffalo, goat and sheep, and cure the cheeses in ripening chambers or subterranean caves, and some add spices (REPEQUAB, 2020). For instance, over 150 cheese varieties are produced in these dairies, highlighting the potential for innovation in cheese production in the state of São Paulo. The tropical climate during hot and rainy summers favors the growth of protein-rich pastures, such as *Capim elefante* (*Pennisetum purpureum*) and *Capim Tanzânia* (*Panicum maximum* cv. Tanzania), while in dry and relatively cold winter, equally rich oat (Avena sp), azevém (*Lolium multiflorum* Lam), sugarcane (*Saccarum officinarum*) and silage are used (EMBRAPA, 2002a; São Paulo, 2017, 2018). The cheese producers from the *Paulista artisanal Cheese Path* are also affiliated to APQA.

##### Porungo Cheese

The *Porungo cheese*, also called *porongo*, *cabacinha, cabaça, porunguinho, nozinho, cabecinha, enforcado* or *pescocinho*, is similar to the *Cabacinha cheese* produced in the state of Minas Gerais, described above. This type of cheese is produced in the southwest of the São Paulo state, mainly in the municipalities of Angatuba, Buri, Campina do Monte Alegre, Itapetininga and Pilar do Sul (Vasek and Filho, 2019; Silva et al., 2021). Porungo is an unripe *pasta filata* cheese, manufactured with raw milk coagulated with commercial rennet and added of fermented whey, collected in the previous day production, that contains the autochthonous microbiota of milk, responsible for the peculiar sensorial characteristics of the cheese, that resemble the mozzarella cheese. *Porungo cheese* producers are not part of the *Paulista Artisanal Cheese Path* or even affiliated to APQA, but commercialization of this product has great economic importance and is a source of income for numerous small producers. These cheeses are sold formally in supermarkets but also informally, directly to consumers or in free markets (Vasek and Filho, 2019).

### Cheeses From the Northeast (States of Alagoas, Bahia, Ceará, Maranhão, Paraíba, Pernambuco, Rio Grande do Norte, and Sergipe)

#### Coalho Cheese

*Coalho cheese* is the most typical artisanal cheese produced in the Northeast region of Brazil, widely consumed by the local population and throughout the country. This is a firm, lightweight yellowish white fresh cheese, prepared with raw milk of cow, buffalo, goat, or sheep and rennet, presenting 35.0–60.0% fat content. It has a slightly salty and acidic flavor and elastic texture and it is used for preparation of the popular “roasted cheese” as it does not melt when heated.

The most relevant *Coalho cheese* producers are located in Batalha, state of Alagoas; Antas, Chapada Diamantina National Park Juazeiro, Feira de Santana and Irecê, state of Bahia; Quixadá and Sobral, state of Ceará; Riachão do Jacuípe, state of Maranhão; Garanhuns and Riacho das Almas, state of Pernambuco; Seridó, state of Rio Grande do Norte; Nossa Senhora da Glória, state of Sergipe and around 50 municipalities in the state of Paraíba (Figure 1).

This cheese has great economic importance for the Northeast region of Brazil, significantly impacting the income of milk suppliers, especially those who lack access to milk processing plants (Brazil, 2001a; Silva et al., 2012; Fontenele et al., 2017; Pernambuco, 2018). It is estimated that about 40–50% of milk production in the region is destined to the production of *Coalho cheese* (Silva et al., 2012; Da Cruz, 2016). In this region, the use of genetically modified animals, such as the F1 hybrid (Dutch/Zebu), is common. These animals present profitable characteristics such as high resistance against lack of rain, and high productivity (Da Cruz, 2016).

#### Manteiga Cheese (Sertão Cheese)

Also called *butter cheese* and produced in many states of the Northeast region of the country, *Manteiga cheese* is soft and has a fat content ranging between 25 and 55%. The taste is light, slightly acidic, and salty, and the color is light yellow (Brazil, 2001b; Leite et al., 2019). Its production consists of coagulating whole or skimmed cow’s milk, draining the curd obtained by acidification, melting and addition of butter or vegetable oil to the melted curd, cooking at 85°C for 15 min and pressing. The butter (*Manteiga de Garrafa*, *Manteiga da Terra* or *Manteiga do Sertão*) used in the manufacture is artisanal as well.

#### Other Artisanal Cheeses of This Region

Other award-winning but not so well characterized goat milk cheeses produced in this region are *Dom Ariano* and *Dom Manelito*, created to honor two famous Brazilian poets (Ariano Suassuna and Manuel Bandeira) and *Cariri cheese*, that honors the religious mysticism of the Cariri region, in the state of Paraíba. *Requeijão Pernambucano*, a soft cheese and *Flor de Mandacaru*, that reminds the French Camembert cheese, are popular cow milk cheeses produced in the state of Pernambuco (Sertão Brás, 2013; Taperoa, 2016).

### Cheeses From the South (States of Paraná, Rio Grande do Sul and Santa Catarina)

The South region of Brazil is characterized by subtropical climate with temperature ranging between 0 (occasionally below 0°C) and 32°C (EPAGRI, 2015). The arrival of European immigrants, mainly Italian and German, to this region in the 19th and 20th centuries had strong influence on the cheese-making culture (Wilkinson et al., 2017). The most prominent artisanal cheeses produced in the South region are the *Serrano*, *Colonial*, and *Kochkäse cheeses*. Less known are the *Diamante* cheese, from the municipality of Major Gercino, *Contestado cheese* from Contestado Valley and *Queijinho* from Itajaí Valley, all located in Santa Catarina state.

#### Serrano Cheese

The *Serrano cheese* is the main type of cheese produced in Serrana Region in the state of Santa Catarina and in Campos de Cima da Serra Region in the state of Rio Grande do Sul (Pretto and Sant’Anna, 2017; Slow Food, 2018). *Serrano cheese* is a semi-fat cheese of medium moisture, made with raw cow milk and ripened for 60 days. Its color is yellowish or straw yellow. It has a compact curd and elastic consistency, tending to the greasiness, and may contain small mechanical and/or propionic eyes, lacking a standard for shape, weight, moisture and salt content (Rio Grande do Sul, 2014, 2016, 2018). The milk comes from mixed breeds, mainly Charolais, Dutch, Devon, Norman, Angus and Hereford, fed on pasture (Slow Food, 2018).

*Serrano cheese* production in the state of Santa Catarina is widespread, with approximately 2,000 producers and 1,600 tons of cheese traded every year, and total gross sales around R$ 21 million (U$ 3.8 million). In Rio Grande do Sul, there are around 1,500 producers that trade 800 tons of *Serrano cheese* per year, with sales of approximately R$ 10 million (U$ 1.8 million; EPAGRI, 2015). In March 2020, the INPI granted the Geographical Indication “*Campos de Cima da Serra*,” in terms of “*Origin Appellation*” to the *Serrano cheese* produced in this location (INPI, 2020).

#### Colonial Cheese

*Colonial cheese* is produced by many rural families in the South of Brazil, especially in Santa Catarina state (Carvalho et al., 2019). Traditionally made from raw cow milk, production had to change to pasteurized milk due to legal requirements. The cheese curd is heated to 30°C and can be added with spices or vegetable products and the minimum ripening period of 10 days is required (Santa Catarina, 2018). The cheeses present square and round shapes, and the weight of each piece varies from 1.0 to 1.2 kg (Fava et al., 2012). The cattle used in milk production belong to Dutch and Jersey breeds, fed on pastures with the addition of corn, sweet potato leaves and forage (Slow Food, 2016a).

#### Kochkäse Cheese

This cheese is an unripened cheese, made from raw or pasteurized milk and the curd is cooked. It is produced in the Itajaí Valley in the state of Santa Catarina, mainly in the cities of Indaial, Timbó, Pomerode, Blumenau, Caminhos do Príncipe, and Joinville. This cheese has a mild flavor and is light yellow (Slow Food, 2016b). Milk comes from Jersey breed, fed with *Capim gramão novo*, *Capim gordura, Catamão branco* and silage. Many families produce *kochkäse* for their own consumption and commercialization, but current health and safety standards have forced some producers to stop producing this cheese (Meisen et al., 2019).

### Artisanal Cheeses From the North

#### Marajó Cheese

The *Marajó cheese* is the most famous artisanal cheese from the North of Brazil. It is an unripened cheese produced for over 200 years in the Marajó archipelago, in the state of Pará, using buffalo milk or a mix of buffalo and cow milk. The buffalo herd in this region is the largest in the country, around 800,000 animals, and among these, 450,000 in state of Pará state (Seixas et al., 2015). The buffalo herd is made up of the *Carabao, Baio, Mediterraneo, Múrrah, Jaffarabadi*, and crossbred breeds (Cassiano et al., 2003). The main milk and buffalo cheese producers are in the municipalities of Soure and Cachoeira do Arari, in the state of Pará (Agência Pará, 2019). There are 60 cheese producers in the Marajó Island, which make 60–100 kg of cheese per day and this activity is very important for the local economy (CEAD, 2014).

The Marajó Island has a rainy tropical climate and an average temperature of 27°C. The rainiest months are January to June and the less rainy ones are September to November (Lima et al., 2005). The climate contributes to the presence of native pastures such as *Capim canarana verdadeira* (*Echinochloa polystachya*) and *Capim quicuio* (*Brachiaria humidicola*), which are used to feed the herd (Meisen et al., 2019).

There are two types of *Marajó cheeses*: one is butter-type, made with whole milk and added butter, and the other is cream-type, made with skimmed milk and cream from skimmed milk. For manufacturing, the curd is drained and washed with water or milk. The product has a light-yellow color and presents slightly acidic and salty flavor and semi-hard consistency (ADEPARÁ, 2013; Vasek and Filho, 2019).

#### Other Artisanal Cheeses From This Region

The *Manteiga* and *Coalho cheeses*, produced in the Northeast region of the country, are also manufactured in Manaus and surroundings, in the Amazonas state, following the same cheese making techniques (Vasek and Filho, 2019).

### Artisanal Cheeses From the Central-West

#### Caipira Cheese

*Caipira cheese* is manufactured in Mato Grosso do Sul state, traditionally recognized as a cattle production state. Nevertheless, in 1980, rural families started producing cheese as an option of income. The majority of producers are located in São Gabriel do Oeste, Corguinho, Rochedo, Jaraguari, Terenos, Ribas do Rio Pardo, Aquidauana, Bandeirantes, Camapuã, Santa Rita do Pardo and Sidrolândia municipalities, besides Campo Grande, the state’s capital. *Caipira cheese* is made with raw, fresh whole cow milk, following historical and cultural tradition of the region of production. The product must be manufactured in the original rural property and must be submitted to 60 days of ripening. The climate of this region is tropical, characterized by hot and rainy weather. The main breeds of cattle used for milk production are Dutch, Swiss-Parda, Schwyz, Jersey, Guernsey, Ayrshire or crossbred, and the feed is based on a mixture of silage, hay, chopped green grass, added with energy and protein concentrates, minerals and vitamins (EMBRAPA, 2002b).

Unlike *Minas artisanal cheese*, the sensorial and physicochemical characteristics of *Caipira cheese* are not well defined, so the producers belonging to the Association of Artisanal Cheese Producers in Mato Grosso do Sul (*Associação dos Produtores de Queijo Artesanal de Mato Grosso do Sul* - AQUEIJART) have recently partnered with the State Agency for Animal and Plant Sanitary Defense (*Agência Estadual de Defesa Sanitária Animal e Vegetal* - AGRAER) and State Secretariat for the Environment, Economic Development, Production and Agriculture (*Secretaria de Estado de Meio Ambiente, Desenvolvimento Econômico, Produção e Agricultura Familiar* - SEMAGRO) in order to establish quality and production parameters, by means of technical-scientific and microbiological studies (Campo Grande News, 2018).

## Safety of Artisanal Cheeses Made with Raw Milk

The microbial communities in cheeses manufactured with raw milk play an important role during ripening. Besides determining the sensorial and physicochemical properties of the final products, they may inhibit the growth of pathogens (Fuka et al., 2013). High humidity and short ripening cheeses are at greater risk of harboring pathogens, in comparison to lower moisture and slower ripening varieties (Ozturkoglu-Budak et al., 2016). In the beginning of ripening, there is a higher prevalence of lactic acid bacteria, mainly *Lactococcus*, *Streptococcus*, and *Lactobacillus*, responsible for the fermentation of sugars and development of the unique sensorial characteristics of artisanal cheeses (aroma, flavor, and texture). Their capability to produce organic acids from sugars during ripening causes pH drop and lowers the oxy-reduction potential. In addition, production of hydrogen peroxide, carbon dioxide, and bacteriocins may inhibit the growth of pathogens (Camargo et al., 2021).

A compilation of studies conducted from 1973 to 2006 in Switzerland, United States, Sweden, Canada, France, Brazil, United Kingdom, Spain, Malta, Scotland, England, and Finland (FSANZ, 2009), based on 84 outbreaks attributed to cheese consumption, concluded that 69% of the outbreaks were associated to raw milk cheeses, while 7.2% were caused by cheeses with no information about heat treatment. Among the outbreaks, two were attributed to cheeses produced in Minas Gerais state, Brazil. However, none of these cheeses fit the category of artisanal cheeses, as defined by the Brazilian regulatory standards (Do Carmo et al., 2002; Brazil, 2019b).

In Brazil, artisanal *Coalho cheese* was incriminated in 55 foodborne disease outbreaks in the Amazonas state between 2005 and 2009. According to the Department of Epidemiological and Environmental Surveillance of Manaus, Amazonas, 14 (25%) of these outbreaks were due to coagulase positive *Staphylococcus aureus*, four (8%) due to *Bacillus cereus*, two (4%) due to *Salmonella* spp. and one due to *Clostridium perfringens*. In one outbreak, both coagulase-positive *Staphylococcus* and *B. cereus* were found. The etiological agent was not determined in the remaining outbreaks (Ruwer et al., 2011).

Even though there are some reports of outbreaks due to consumption of raw milk and raw milk cheeses around the world, accurate and official information on this issue is lacking in Brazil. Data from the National Health Surveillance Agency of the Brazilian Ministry of Health indicate that milk and dairy products were responsible for 2.75% of the foodborne outbreaks reported in the 2000–2018 period (Brazil, 2019a; Finger et al., 2019). Even considering that the type of dairy product associated with the reported outbreaks is unknown and that the number of outbreaks is possibly underreported, the relevance of artisanal cheeses prepared with raw milk as causes of foodborne diseases should not be ignored.

One of the major concerns in dairy products made with unpasteurized milk are *Brucella* spp. and *Mycobacterium bovis. Brucella* spp. causes brucellosis, a zoonosis that can be transmitted from animals to humans and vice versa. In cattle, this disease can cause abortion and congenital related problems, while in humans it is usually manifested as a general infection. This pathogen can be present in the mammary gland of infected animals and eliminated through milk. Brucellosis presents economic losses due to the reproductive problems caused by the disease in cattle (Paulin and Ferreira Neto, 2008). Another important zoonotic disease is tuberculosis caused by *M. bovis*, which contributes to the development of nodular lesions in tissues or organs of the animal, and causes abortion, fever, drop in milk production and weight reduction, which can lead to the death or slaughter of animals. In humans, the disease can be transmitted through the consumption of unpasteurized milk and dairy products, the consumption of uncooked meat or by contact with the infected animal and may present symptoms such as fever, weight loss, lung problems, cough, shortness of breath, among others (Kuria, 2019). The Brazilian regulations, by means of the National Program for the Control and Eradication of Brucellosis and Animal Tuberculosis, set requirements for the control of *Brucella* spp., *M. bovis* and pathogens in dairy products manufactured with raw milk, establishing that herds must be certified as brucellosis and tuberculosis free (Brazil, 2019c).

*Staphylococcus. aureus* is another relevant microbial pathogen in unpasteurized milk, as it causes mastitis, an infectious process that affects the mammary gland and causes changes in the secretion and composition of milk, resulting in great economic losses in milk production. *Staphylococcus aureus* in contaminated milk can be transmitted to dairy products, and cause intoxication, through production of enterotoxins. In addition, this pathogen is also present in cheese due to improper handling (Dias, 2007; Neto and Zappa, 2011; Dittmann et al., 2017). High counts of *S. aureus* are the main nonconformities found in Brazilian artisanal cheeses, but there is a general lack of data related to staphylococcal intoxication in these products (Finger et al., 2019; Camargo et al., 2021). It is possible that microbial interactions in the cheese matrix suppress synthesis of enterotoxins or the strains that contaminate these products are not enterotoxin producers. Additional studies are needed to evaluate the behavior of native *S. aureus* strains in these products.

Other relevant pathogens that can be found in artisanal cheeses are *Salmonella* and *Listeria monocytogenes*. These pathogens may originate from raw materials (milk) or from the factory environment, especially from the processing area, including equipment, personnel or cross contamination between finished products and raw materials (Williams, and Withers, 2010; McIntyre et al., 2015; Muhterem-Uyar et al., 2015; Aragon-Alegro et al., 2021). The occurrence of *Salmonella* and *L. monocytogenes* in cheeses with higher humidity is more common, suggesting that lower water content could be less favorable to their survival. Also, low pH values, low water activity (a_w_) and the presence of lactic acid bacteria that have antimicrobial activity may decrease or eradicate the presence of these pathogenic microorganisms, and probably because of that, the studies conducted so far indicate that prevalence of *Salmonella* spp. and *L. monocytogenes* in Brazilian artisanal cheeses is low (Table 2).

**Table 2:** Summary of studies on pathogenic bacteria in Brazilian artisanal cheeses.

Type of cheese	Number of tested samples	Results highlights	Reference
**Coagulase positive *Staphylococcus* (CPS)**			
*Canastra*	10	Seven samples presented counts of *S. aureus* above 4.8 log CFU g^−1^ with 5 days of ripening. Nine *S. aureus* isolates were positive for at least one toxin (SEA, SEB, SEC, SED and TSST-1)	Borelli et al., 2006
*Serro*	100	Counts of CPS were lower than 3 log CFU g^−1^ after 15 days of ripening in the rainy season and 45 days of ripening in the dry season^*^. SEB and SEC were found in some samples of cheese.	Cardoso et al., 2013
*Araxá*	30	28 samples presented CPS counts above 3 log CFU g^-1^^*^ (no information about ripening time)	Souza et al., 2015
*Serro*	53	40 samples presented CPS counts above 3 log CFU g^-1^^*^ (no information about ripening time). None of the isolates produced SEA, SEB, SEC, SED and SEE nor had SE related genes	Andretta et al., 2019
*Canastra*	78	33 samples presented CPS counts above 3 log CFU g^-1^^*^ after 22 days of ripening	Campos et al., 2021
***Listeria* spp. and *L. monocytogenes***			
*Coalho* (Manaus, AM)	58	*Listeria* sp. was detected in 2 samples	Ramos and Costa, 2003
*Canastra*	32	*Listeria* sp. was not detected	Dores et al., 2013
*Serro* (17 days of ripening)	256	*L. monocytogenes* was not detected	Martins et al., 2015
*Coalho* (Pernambuco)	60	*Listeria grayi* detected in 2 samples	Aragão et al., 2019
*Canastra*	78	*L. monocytogenes* detected in 1 sample.	Campos et al., 2021
**Pathogenic *Escherichia coli***			
Raw milk cheeses from Paraná, São Paulo, Minas Gerais, Mato Grosso do Sul and Bahia	147	*E. coli* was found in 28 cheeses. One among 39 *E. coli* isolates was positive for *eae* gene, and negative for *bpf* and *efa1/lifA* genes, being classified as atypical EPEC (aEPEC).	Campos et al., 2017
*Serrano* (Santa Catarina)	109	22 among 109 *E. coli* isolates presented the *eae* (EPEC), *st* and *lt* (ETEC) or *aggR* (EAEC) genes	Parussolo et al., 2019
***Salmonella* spp.**			
Coalho (Pernambuco)	127	*Salmonella* spp. was detected in 7 samples	Duarte et al., 2005
*Serro*	100	*Salmonella* spp. was not detected	Cardoso et al., 2013
*Coalho* (*artisanal and non-artisanal samples*, Northeast)	104	*Salmonella* spp. was detected in 1 artisanal cheese sample	Sousa et al., 2014
*Serro*	256	*Salmonella* spp. was detected in 1 sample	Martins et al., 2015
*Minas artisanal* (Montes Claros, MG)	18	*Salmonella* spp. was detected in 2 samples	Pinto et al., 2016
*Canastra*	78	*Salmonella* spp. was not detected	Campos et al., 2021
***Brucella* spp.**			
*Minas artisanal* (4 and 8 days of ripening) (Serro, MG)	55	*Brucella* spp. was detected in 17 samples	Silva et al., 2018

* Counts of CPS must be lower than 3 log CFU.g-1 according to Minas Gerais (2008).

Besides the above cited microorganisms, other pathogens that have been associated to outbreaks caused by cheeses manufactured with unpasteurized milk in other parts of the world are Shiga Toxin-producing *E. coli* (Deschênes et al., 1996), *Salmonella* Muenster (van Cauteren et al., 2009), *Salmonella* Typhi (FSANZ, 2009), *Brucella* spp. (Linnan et al., 1988; FSANZ, 2009), *Campylobacter* (Gould et al., 2014), and *Campylobacter jejuni* (Oliver et al., 2009). These etiological agents have caused outbreaks of different intensity and severity, showing that they deserve the attention of those responsible for the safety of artisanal cheeses.

Some studies have evaluated the occurrence of pathogenic microorganisms in different types of Brazilian artisanal cheeses. Silva et al. (2018) evaluated the presence of *Brucella* spp. in 55 samples of Serro artisanal Minas cheese and observed that 17 tested positive. The study was conducted with samples ripened for 4 and 8 days only, using a culture independent method (Nested-PCR). Additional studies with cheeses ripened for longer periods, as required by state regulation for this particular type of cheese, are still pending.

Dores et al. (2013) evaluated the effect of the ripening temperature (8 and 25°C) on the counts of pathogenic and indicator bacteria in *Canastra* cheeses. They observed that ripening at 25°C for 22 days was sufficient to decrease the counts of total coliforms, *Escherichia coli* and *Staphylococcus aureus* to the levels required by the legislation (< 1,000 CFU g^−1^ for *E. coli* and coagulase positive staphylococci), while ripening at 8°C required 64 days to reach these levels. None of the tested cheeses presented *Listeria monocytogenes* or *Salmonella* spp. Lower values of a_w_ and higher pH and sodium chloride were detected in cheeses ripened at 25°C, suggesting that these characteristics may have had a positive effect on the control of pathogens. In a similar study conducted by Martins et al. (2015) with *Serro cheese* ripened at room temperature (25 ± 4°C) and under refrigeration (8 ± 1°C), the authors observed that ripening at room temperature for 17 days was the ideal condition to reduce the counts of *S. aureus* to safe limits (< 1,000 CFU g^−1^). *Listeria monocytogenes* was not detected in the 256 tested cheese samples, but *Salmonella*, present in one initial sample, was no longer detected after 22 days of ripening.

Mata et al. (2016) evaluated the effect of “*pingo*” collected in the *Serro* region on the survival of *Listeria* sp. during ripening of cheeses produced in laboratory conditions. Cheeses were prepared with raw milk experimentally contaminated with *Listeria innocua* ATCC 33090 (10 CFU ml^−1^). Results indicated that *L. innocua* was not eliminated even after 60 days of ripening at 30°C, showing that the physicochemical changes and activity of the competitive microbiota during ripening may not be enough to guarantee the absence of *L. monocytogenes* in the final product under tested conditions.

Campagnollo et al. (2018) conducted a quantitative risk assessment of *L. monocytogenes* in semi-hard cheeses prepared with raw milk experimentally contaminated with 6 log CFU ml^−1^ of *L. monocytogenes* and ripened up to 22 days at 22 ± 2°C. The authors concluded that these cheeses presented lower risk of listeriosis than a soft cheese produced with pasteurized milk containing 1 log CFU ml^−1^ of *L. monocytogenes*, observing that the longer the ripening time the lower the risk. This study reinforced that mitigation of listeriosis risk is related to the cheese ripening period, when pH decreases, sodium chloride concentration increases, a_w_ decreases and interactions with lactic acid bacteria control the survival of *L. monocytogenes*.

Recently, Campos et al. (2021) evaluated safety indicators and pathogens from Canasta cheeses during the production process, including ripening. They confirmed that 22 days of ripening are sufficient to control the populations of hygiene indicator microorganisms (total coliforms, coagulase-positive *Staphylococcus* and *E. coli*) in *Canastra cheese* samples in accordance with the levels established by the current regulations, provided that the producers adopt good manufacturing practices. *Salmonella* was not detected in the study, but *L. monocytogenes* was detected in one sample, reinforcing the importance of the good hygiene and manufacturing practices.

Several studies have evaluated the presence of coagulase positive *Staphylococcus* (CPS), *Listeria* and *L. monocytogenes*, pathogenic *E. coli*, *Salmonella* and *Brucella* spp. in artisanal cheeses manufactured in Brazil. Results of these studies are summarized in Table 2.

## Good Hygiene and Manufacturing Practices

Brazilian regulations on artisanal cheese production indicate that the good hygiene and manufacturing practices must start at the farm level, i.e., cattle feeding, vaccination, and milking (Paulin and Ferreira Neto, 2008; Brazil, 2013, 2018, 2019b; Minas Gerais, 2017). Brazil has extensive legislation on hygiene and manufacturing practices for artisanal cheeses. Over the years, there have been major changes such as allowing marketing of raw milk cheeses provided they are ripened for 60 days, through a federal resolution (Brazil, 2000). Years later, a state normative allowed Minas artisanal cheeses such as *Canastra cheese* to be marketed with less than 60 days of ripening, reducing this requirement to 22 days (Minas Gerais, 2013). The reduction in the ripening period was based on a study that concluded that 22 days are enough to ensure safety as long as the cheeses are ripened at room temperature (Dores et al., 2013). The mentioned study was based on counts of hygiene and food safety indicator microorganisms only; thus, additional studies are vital to validate shorter ripening times, including the detection of hazards, such as *Brucella, Mycobacterium*, and *Staphylococcus* enterotoxins.

One of the most important changes in the Brazilian legislation regarding artisanal products of animal origin occurred in 2018, when the ARTE Seal (ARTE, short for *artesanal* – artisanal in Portuguese) was created. Cheeses with the ARTE Seal can be marketed interstate without restrictions, provided they are inspected by State or Federal Agencies (Brazil, 2018). The Brazilian scientific sector played a relevant role in defining the minimum ripening period for artisanal cheeses, with the involvement of many researchers across the country. These scientists highlighted the importance of the good manufacturing practices in the production chain and played an important role in disseminating this knowledge to cheese producers around the country.

The milking process is a critical control point. Guidelines of EMATER (State Technical Assistance and Rural Extension Company) determine that before milking, udders must be washed with chlorinated water containing 2–3 ppm of free chlorine and disinfected properly by pre-dipping with a chlorinated solution (50–100 mg L^−1^). After milking, the udders must be disinfected with iodine solution at 20–30 mg L^−1^ or another recommended disinfectant. Animals with mastitis should be milked last and their milk cannot be destined for cheese production. Milk intended for artisanal cheese production should be filtered in 10–16 mesh filters before entering the cheese making facility and again using 60–90 mesh filters before entering the production tank (EMATER, 2009).

Cheese production sites must be well structured and maintained. The quality and safety of the water must be controlled, and corrals and milking rooms must be well-finished, with easy disposal of water and organic waste. Walls must be painted with washable paint and floors must be sturdy, waterproof and from non-slippery material. Walls, floors, utensils, and equipment should be subjected to cleaning and disinfection with chlorinated solution, before and after milking. The cleaning of milk reception and raw material storage areas must be monitored, and manufacturing areas must be adequately ventilated (SEBRAE, 2015).

Workers at the cheese production sites must keep updated health certificates and wear clean and appropriate clothing, rubber boots, masks, and hat. Workers with health problems (cuts, wounds, cold, among others) cannot have access to the cheese manufacturing area. Hand washing with disinfectants is mandatory, before and after milking. Bad practices, such as smoking, sneezing, coughing, scratching the head, etc., and visitors must be avoided (EMATER, 2009; SEBRAE, 2015).

## Animal Health Protection

The Brazilian Ministry of Agriculture, Livestock and Food Supply and state agencies of animal health protection require vaccination for brucellosis by means of the National Program for the Control and Eradication of Brucellosis and Animal Tuberculosis (MAPA, 2019). Vaccination against brucellosis is mandatory for females in ages between 3 and 8 months. Vaccination against foot-and-mouth disease must be applied directly by the health authorities of each state. The vaccine against symptomatic anthrax must be applied to all animals on the third month of age and must be repeated every 6 months until 2 years of age. Another important vaccination is against rabies that should be applied annually, especially in outbreak regions when much of the herd can be affected by the disease (MAPA, 2019). There are some vaccines that can be used to control some animal diseases such as botulism, clostridium disease, leptospirosis, and infectious bovine rhinotracheitis (IBR), bovine viral diarrhea (BVD), mastitis, campylobacteriosis and colibacillosis (EMBRAPA, 2019b).

An effective feeding system is also relevant for animal health protection. It must provide energy, protein, minerals, and vitamins to meet the nutritional needs of each category of animal and at each stage of the life cycle of animals in the herd (Salman et al., 2011). The planning of a balanced diet is an indispensable strategy for a positive impact on the economy and production in the livestock sector (EMBRAPA, 2003).

## Perspectives for Improvements

Brazil is a country with continental dimensions and hence with a great diversity of climate, vegetation, topography, and culture that directly reflects the diversity of cheeses produced in the country, as indicated in this review. The popularity of artisanal cheeses in the national market has been growing. In terms of flavor, these cheeses have already proven their attributes and value in international contests. The regulation for artisanal cheese production is numerous, and producers, particularly the small ones, consider it too rigorous and sometimes confusing and not well accepted or understood. The recent improvements in the regulations, at local, state, and national levels, that revised old and obsolete laws, have contributed to combat clandestinity, bringing significant economic turn over for producers. The role of consumers demanding better quality and safety is also important. These actions, alongside with technical qualification of producers and incentives for research projects, will contribute to elevate Brazilian artisanal cheeses to worldwide recognition. In this sense, producers’ associations play an important role, as they protect the identity of the products, promote educational actions that improve production practices and assist in the proper publicity of artisanal products throughout the national territory (APROCAN, 2021). This process of cooperativism is gaining strength, being a reasonable solution to increase the market share of these products and improve their overall quality.

The challenges to assure absence of pathogens in Brazilian artisanal cheeses are no different than those in similar cheeses produced elsewhere: they are attributed to the use of unpasteurized milk and to disruptions in the production/trade chain regarding the failure in good hygiene and good manufacturing practices. The good hygiene practices must be adopted at all stages, from proper vaccination of the herd to milking and manufacturing up to consumption, in order to guarantee microbiological safety and avoid public health problems. For the effective application of these practices, the proper training of cheese producers and food handlers is mandatory.

One issue that seems to be unique in the country is the minimal ripening time necessary to guarantee microbiological safety of artisanal cheeses produced with raw milk. Brazilian legislation, that follows international norms, require a minimum of 60 days of ripening, but recent state regulations allow shorter ripening time such as 14–22 days, depending on the geographical origin of the cheese. These new regulations were based on local studies that evaluated hygiene microbiological indicators, primarily focusing on counts of coliforms, *E. coli*, and *S. aureus* and the detection of *Salmonella* and *L. monocytogenes* in the final product. Some studies have confirmed the safety of these products regarding these microbiological criteria, but additional research, including the detection of other microorganisms, such as *Brucella* spp. and *Mycobacterium* sp., as well as *Staphylococcus* enterotoxins in the product, would increase information on the safety of these cheeses. It is important to point out that there is a lack of data related to the detection of microbial pathogens in artisanal cheeses as well as on beneficial microbiota, especially considering the huge diversity of artisanal cheeses produced in the nation.

Most of the artisanal cheeses produced in the country have been characterized by culture dependent methods and traditional chemical approaches. Besides including the diverse types of artisanal cheeses produced nationwide, it is important that new studies use state of the art genomic and metabolomic approaches that could reveal the singularities of each producing region, helping define the unique microbiological and chemical profiles of these products. Finally, studies considering the microbial interaction in the cheese making environment, including the cheese making facility and the product during ripening, will reveal the kinds of interactions that take place in products that have desirable safety and sensorial features. For instance, a study conducted with cocoa beans has revealed the role of quorum sensing and cross-feeding in shaping microbial succession during fermentation, as discussed by Almeida et al. (2020).

Recently, an initiative known as Brazilian Artisanal Cheese Research Network (REPEQUAB – Rede de Pesquisas em Queijos Artesanais Brasileiros^1^) was created with the aim of integrating scientists from all Brazilian producing regions in order to promote the exchange of knowledge, samples, databases, and especially to stimulate collaborative research to solve regional and national issues related to artisanal cheese production. The network has already connected 70 researchers, and several collaborative investigations are ongoing, especially in the Canastra region in Minas Gerais and in the state of Sao Paulo. Future meetings aiming to discuss the advancements in the field and to build new collaborative investigations will drive quality and safety improvements in artisanal cheese production in the country.

## Author Contributions

UP planned the manuscript. AP and GC wrote and revised the drafts of the manuscript. AP, GC, NP-F, BF, and UP contributed to write and revise the drafts of the manuscript. UP and BF edited the manuscript. All authors contributed to the article and approved the submitted version.

### Conflict of Interest

The authors declare that the research was conducted in the absence of any commercial or financial relationships that could be construed as a potential conflict of interest.

## Footnotes

^1^http://repequab.com.br/
